# Remarkable response to third-generation EGFR-TKI plus crizotinib in a patient with pulmonary adenocarcinoma harboring EGFR and ROS1 co-mutation: a case report

**DOI:** 10.3389/fonc.2024.1357230

**Published:** 2024-02-27

**Authors:** Zhiming Wu, Zelin Zhang, Dongdong Zhang, Zengyan Li

**Affiliations:** ^1^ Department of Orthopedics, Xiangyang No. 1 People’s Hospital, Hubei University of Medicine, Xiangyang, China; ^2^ Department of Oncology, Xiangyang No. 1 People’s Hospital, Hubei University of Medicine, Xiangyang, China

**Keywords:** non-small cell lung cancer, EGFR, ROS1, tyrosine kinase inhibitors, co-mutation

## Abstract

**Background:**

Driver oncogene mutations, such as c-ros oncogene 1 (ROS1) and epidermal growth factor receptor (EGFR) were previously believed to be mutually exclusive in non-small cell lung cancer (NSCLC). Only sporadic cases of ROS1 and EGFR co-mutations have been reported. Hence, appropriate treatment options for these patients are still controversial.

**Case presentation:**

A 48-year-old female patient presented at our hospital complaining of a persistent cough that had been ongoing for a month. A chest computed tomography showed a mass in the left lung along with hilar and mediastinal lymphadenopathy. Pathological analysis of bronchoscopic biopsy and lung mass puncture confirmed the presence of lung adenocarcinoma. The patient was diagnosed with stage IIIC left lung adenocarcinoma with a clinical stage of cT2N3M0. Next-generation sequencing analysis conducted at both puncture sites revealed an EFGR 19 deletion mutation combined with ROS1 rearrangement. The lung mass exhibited a higher mutation abundance. Treatment with a combination of third-generation EGFR tyrosine kinase inhibitors (TKIs) and crizotinib yielded satisfactory results. During the follow-up period, the mass significantly reduced and almost disappeared.

**Conclusion:**

The co-mutation of EGFR and ROS1 is a rare phenomenon. Nevertheless, the combination of EGFR-TKI and crizotinib treatment appears to hold promise in providing positive results for patients, with manageable side effects. This therapeutic approach has the potential to enhance patients’ overall prognosis.

## Introduction

Lung cancer is still the leading cause of cancer−related deaths globally, and non-small-cell lung cancer (NSCLC) accounts for approximately 85% of all lung cancers. Identifying driver mutations in lung cancer has paved the way for personalized targeted treatments. Therefore, screening patients with lung cancer for oncogenic drivers and administering them with appropriate targeted therapies are of significance (1).

The *EGFR* gene is located in the 12–14 region of the short arm of chromosome 7. EGFR mutations are common in patients with NSCLC (2), especially among East Asian female patients with lung adenocarcinoma (3). Although various mutations have been detected, almost 90% are located in exons 19 and 21 of the *EFGR* gene. These include the deletion of amino acids 746–750 in exon 19 [del (746–750)] and a leucine-to-arginine substitution at position 858 in exon 21 (L858R) (4). In 2007, Rikova identified SLC34A2-ROS1 and CD74-ROS1 rearrangements in NSCLC cell lines and patients’ tumors (5), establishing oncogenic c-ros oncogene 1 (ROS1) rearrangements as a therapeutic target in lung cancer. Oncogenic ROS1 gene fusion was found in a distinct subpopulation (about 1 1%–2%) of patients with NSCLC. These patients were typically characterized by adenocarcinoma histology, young age, and a nonsmoking history (6).

Previous studies indicated that ROS1 rearrangements and EGFR mutations were mutually exclusive (5, 6). However, in this study, we presented the case of a patient who exhibited co-mutations in ROS1 and EGFR, and achieved significant remission through the oral administration of two corresponding TKIs. The patient experienced tolerable side effects during the treatment. This case report aimed to provide feasible treatment options for patients with similar co-mutations.

## Case presentation

In February 2023, a 48-year-old woman was admitted to our hospital with a 1-month history of cough. She had no previous history of smoking or alcohol consumption and no family history of malignancy. A chest computed (CT) scan tomography revealed a left lower lobe mass measuring approximately 3.6 × 3.5 cm^2^, along with multiple enlarged mediastinal and hilar lymph nodes.

**Figure 1:**
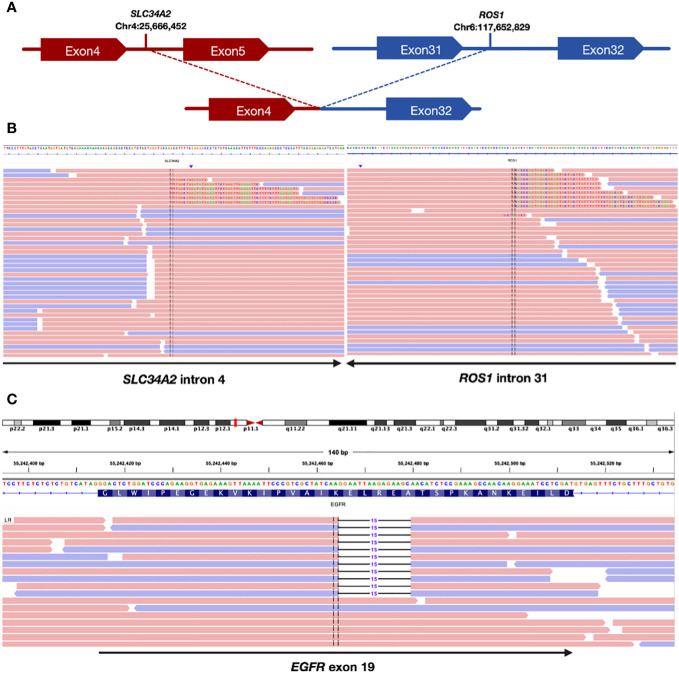
Sequencing assay showed positive ROS1 fusion and EGFR mutation. **(A)** Schematic indicating the fusion between the SLC34A2 gene exon 4 and ROS1 gene exon 32. **(B)** Split-read alignment visualizing the breakpoint of the fusion between SLC34A2 and ROS1 sequences. **(C)** Sequencing read alignment for EGFR exon 19, showing a potential deletion mutation.

A pathological examination was conducted to further clarify the nature of the lesion. The biopsy, performed using endoscopic ultrasound-guided transbronchial needle aspiration, confirmed the presence of lung adenocarcinoma. The patient was clinically diagnosed with stage IIIB lung adenocarcinoma, with a clinical stage of cT2bN3M0(he 8th TNM staging system). Additionally, next-generation sequencing (NGS) examination revealed an ROS1 fusion (SLC34A2) (**Figures 1A, B**) with an abundance of 1.39% and an EGFR exon 19 mutation (**Figure 1C**) with a mutation abundance of 0.61%. The tumor showed a programmed cell death-ligand1(PD-L1) expression of 60%. We performed a CT-guided puncture biopsy of the lung mass with the patient’s consent to improve accuracy and develop an effective treatment plan. The biopsy confirmed the pathological diagnosis of lung adenocarcinoma. The results of the second NGS test were consistent with the previous test results (**Figure 1**), except for a higher mutation abundance observed in the EGFR mutation (14.38%) and the ROS1 fusion mutation (5.93%).

**Figure 2:**
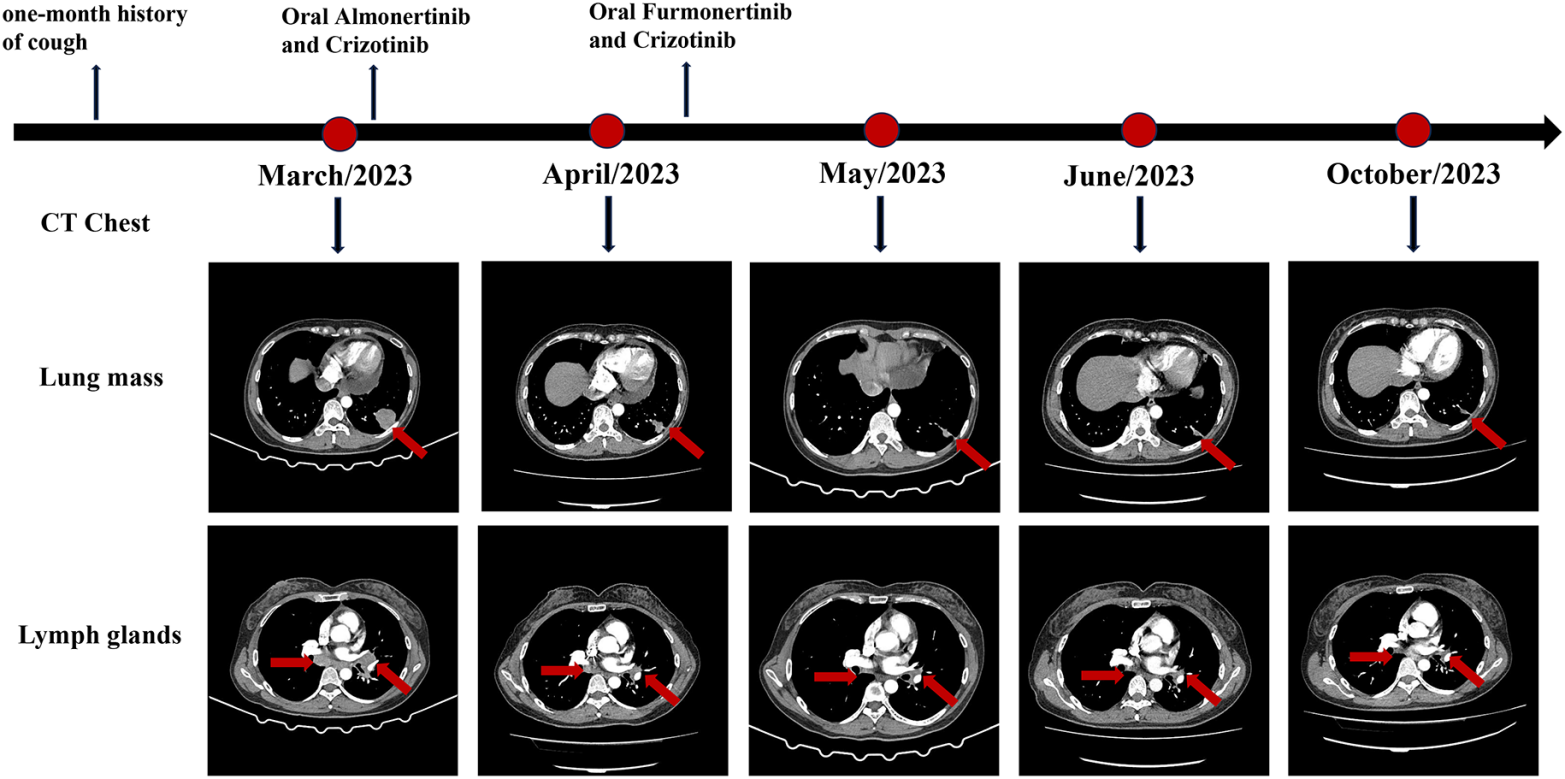
CT scan of the patient throughout the whole course of diagnosis and treatment.

Upon confirming the diagnosis, the patient was administered almonertinib and crizotinib orally. A significant reduction in the primary mass and all lymph nodes was observed after 1 month, and the efficacy was evaluated as a partial response based on the Response Evaluation Criteria In Solid Tumors. However, the patient experienced significant elevations in the levels of creatine kinase (CK) (552.30 U/L; reference range: 40–200 U/L), CK isoenzyme (CK-MB) (58.4 U/L; reference range: 0–25 U/L), and lactate dehydrogenase (LDH) (350.4 U/L; reference range: 120–250 U/L). Fortunately, the patient did not have any symptoms such as muscle soreness. The increase in CK and CK-MB levels aligned with the common adverse reactions of almonertinib (35.5%), indicating that these abnormalities were likely the side effects of almonertinib rather than of crizotinib. Considering the effectiveness of this treatment, we replaced another EGFR-TKI, furmonertinib. As predicted, the levels of CK and CK-MB gradually returned to normal without using any drugs following the discontinuation of almonertinib. The replacement of targeted drugs did not impact the patient’s response to treatment. Continuous monitoring of chest CT scans revealed a gradual reduction in tumor size, almost leading to complete disappearance. The patient has been undergoing treatment for 7 months and currently has stable disease. During subsequent treatment, the patient experienced only a tolerable first-degree rash reaction. The detailed diagnosis and treatment process is shown in **Figure 2**.

## Discussion

Previous studies have indicated that driver genes are usually mutually exclusive. However, technological advancements have increased the focus on NSCLC cases with dual-positive or even triple-positive driver genes. This is due to the growing feasibility of NGS in clinical practice, which allows for high-sensitivity assays with broad-spectrum genomic targets. The Lung Cancer Mutation Consortium project found that 5% of driver alterations in lung adenocarcinoma were double or multiple mutations (7).

At present, ROS1 rearrangements and EGFR mutations have been found to coexist in a small subgroup of patients with NSCLC (8). In a study by Lin et al., out of 220 ROS1-rearranged patients with NSCLC, only 1 patient was identified with both ROS1 fusion and EGFR-activating mutation (9). Another study reported concurrent genomic alterations with ROS1/EGFR co-occurrence observed in 3.17% of patients with EGFR-mutated NSCLC and 16.67% with ROS1-positive NSCLC (8). Additionally, Yanjiao Mao et al. found concomitant ALK/ROS1 fusion genes in 3.1% of patients with EGFR-mutated lung adenocarcinoma (10). The variation in concurrent mutation rates and associated partners could be attributed to the differences in detection methods, sample types, ethnic backgrounds, and tumor heterogeneity. However, the specific incidence still needs to be confirmed in future studies. This case report presented a case of EGFR and ROS1 common mutations combined with high PD-L1 expression and outlined our proposed treatment plan.

Significant progress has been made in the medical therapy of lung cancer in the last decade, largely due to the introduction of targeted therapy. Currently, tyrosine kinase inhibitors (TKIs) are considered a standard treatment for patients with advanced non-small cell lung cancer (NSCLC) who have specific gene mutations. These TKIs have significantly improved the prognosis for these patients (11). For patients with “classic” EGFR mutations (Ex19Dels and L858R), third-generation EGFR-TKIs have been shown to significantly increase progression-free survival and overall survival. Crizotinib, which has been extensively studied in both prospective and retrospective clinical trials (12), has demonstrated remarkable effectiveness in patients with advanced NSCLC carrying ROS1 alterations (13). It was approved by the US Food and Drug Administration and the European Medicines Agency in 2016.

Targeted therapy remains the preferred treatment for patients with co-mutations; however, determining the appropriate TKI for use still poses a challenge (10). Only sporadic reports exist on the selection of targeted drugs due to the rarity of EGFR and ROS1 co-mutations (14). Among these rare reports, there were cases of obvious response to EGFR TKI but ineffective to crizotinib (10, 15), and cases of response to crizotinib but ineffective to EGFR TKI (10, 16). Among patients with EGFR-activating mutations, approximately 70%–80% respond to EGFR-TKIs, whereas the remaining 20%–30% show a poor response, of which 7%–12% progress despite treatment with EGFR-TKIs, suggesting a risk for primary or intrinsic resistance (17).

Concomitant genetic alterations were found to be associated with primary resistance in patients with EGFR-mutated NSCLC (17, 18). These co-existing mutations included T790M and KRAS and the activation of the downstream PI3K-Akt due to factors such as PTEN loss or PIK3CA mutation (17). The mechanism of co-mutation is not clear. Research suggests that two driver alterations can develop within the same clone of tumor cells and may potentially work together during cancer development (19). Two hypotheses have been proposed to explain the coexistence of multiple oncogenic drivers in NSCLCs. Increasing evidence has shown that genetic instabilities in cancer cells lead to genetic and phenotypic heterogeneities within tumors, indicating that different genetic alterations may occur in different tumor cells rather than in a single clone (16). The interactions between EFGR and ROS1 in patients with co-mutations are not well understood. Previous case reports have tried to use EGFR-TKI or crizotinib alone, and no reports exist exploring the combination of EGFR-TKI and crizotinib.

Current genetic testing methods cannot determine which oncogene aberration is more dominant in the same sample. The results of genetic testing may provide us with some clues. The relative levels of phospho-EGFR can be used to predict the efficacy of targeted treatment in EGFR mutants (20). In addition, gene abundance can also be used as a predictor of targeted therapy (21). The rate of ROS1 rearrangement in lung cancer is relatively low, and limited research exists exploring the relationship between ROS1 mutation abundance and lung cancer prognosis. In NSCLCs, CD74–ROS1 is the most common ROS1 fusion (~44%), followed by EZR–ROS1 (16%), SDC4–ROS1 (14%), and SLC34A2–ROS1 (10%) (12). Some scholars have found that SDC4 and SLC34A2 are more likely to coexist with EGFR mutations and ALK translocations (8). In our case, NGS analysis of mediastinal lymph nodes and the primary lung mass indicated the presence of SLC34A2-ROS1 rearrangements along with an EGFR 19 Del mutation, with variations in gene mutation abundance. This phenomenon indicated tumor heterogeneity, which might lead to different dominant targets. Therefore, for this patient, we chose third-generation EGFR-TKI combined with crizotinib, which yielded positive results.

Previous studies have indicated that PD-L1 expression decreases in EGFR-mutated NSCLC compared with EGFR wild-type NSCLC (22). This is because activating the EGFR pathway can induce PD-L1 expression in NSCLC cell lines (23). Currently, data on the expression of PD-L1 in patients with common mutations are lacking. In our case, both tests revealed high expression of PD-L1. The impact of PD-L1 expression on the efficacy of targeted therapy remains controversial. Only patients without actionable driver mutations can benefit from immunotherapy-based treatment, whereas the efficacy of ICIs in patients with actionable driver mutations, regardless of PD-L1 expression, has been limited (24, 25). However, immunotherapy combined with chemotherapy may provide new hope for patients resistant to the third−generation EGFR−TKIs (26). Further research is needed to determine whether ICIs can benefit patients with co-mutations in the future.

In summary, it is necessary to perform full gene detection in NSCLC patients. Dealing with overlapping mutations in driver genes poses significant challenges when tailoring individualized therapy for patients with NSCLC. The role of EGFR and ROS1 inhibitors in patients with dual-positive mutations is unclear in current and previous studies. Further investigation is needed to understand the molecular mechanisms that determine responsiveness to EGFR-TKIs and ROS1-TKIs, as well as potential combination or sequential treatment strategies for this specific subgroup with co-alterations. The case reported in this study provides valuable insights for future research in this area.

## Data availability statement

The clinical data supporting the conclusions of this article will be made available by the authors.

## Ethics statement

This study was approved by the Ethics and Scientific Committeeof Hubei University of Medicine with approval number XYY2021002. Written informed consent was obtained from the individual for the publication of any potentially identifiable images included in this article.

## Author contributions

ZW: Writing – review & editing. ZZ: Writing – review & editing. DZ: Writing – original draft. ZL: Writing – original draft, Writing – review & editing.
